# Healthcare workers' attitudes to working during pandemic influenza: a qualitative study

**DOI:** 10.1186/1471-2458-9-56

**Published:** 2009-02-12

**Authors:** Jonathan Ives, Sheila Greenfield, Jayne M Parry, Heather Draper, Christine Gratus, Judith I Petts, Tom Sorell, Sue Wilson

**Affiliations:** 1 Centre for Biomedical Ethics, The University of Birmingham, Birmingham, UK; 2 Primary Care Clinical Sciences, The University of Birmingham, Birmingham, UK; 3 Department of Public Health, Epidemiology & Biostatistics, The University of Birmingham, Birmingham, UK; 4 Geography, Earth and Environmental Sciences, The University of Birmingham, Birmingham, UK; 5 Centre for the Study of Global Ethics, Edgbaston, Birmingham, UK

## Abstract

**Background:**

Healthcare workers (HCWs) will play a key role in any response to pandemic influenza, and the UK healthcare system's ability to cope during an influenza pandemic will depend, to a large extent, on the number of HCWs who are able and willing to work through the crisis. UK emergency planning will be improved if planners have a better understanding of the reasons UK HCWs may have for their absenteeism, and what might motivate them to work during an influenza pandemic.

This paper reports the results of a qualitative study that explored UK HCWs' views (n = 64) about working during an influenza pandemic, in order to identify factors that might influence their willingness and ability to work and to identify potential sources of any perceived duty on HCWs to work.

**Methods:**

A qualitative study, using focus groups (n = 9) and interviews (n = 5).

**Results:**

HCWs across a range of roles and grades tended to feel motivated by a sense of obligation to work through an influenza pandemic. A number of significant barriers that may prevent them from doing so were also identified. Perceived barriers to the ability to work included being ill oneself, transport difficulties, and childcare responsibilities. Perceived barriers to the willingness to work included: prioritising the wellbeing of family members; a lack of trust in, and goodwill towards, the NHS; a lack of information about the risks and what is expected of them during the crisis; fear of litigation; and the feeling that employers do not take the needs of staff seriously. Barriers to ability and barriers to willingness, however, are difficult to separate out.

**Conclusion:**

Although our participants tended to feel a general obligation to work during an influenza pandemic, there are barriers to working, which, if generalisable, may significantly reduce the NHS workforce during a pandemic. The barriers identified are both barriers to willingness and to ability. This suggests that pandemic planning needs to take into account the possibility that staff may be absent for reasons beyond those currently anticipated in UK planning documents. In particular, staff who are physically able to attend work may nonetheless be unwilling to do so. Although there are some barriers that cannot be mitigated by employers (such as illness, transport infrastructure etc.), there are a number of remedial steps that can be taken to lesson the impact of others (providing accommodation, building reciprocity, provision of information and guidance etc). We suggest that barriers to working lie along an ability/willingness continuum, and that absenteeism may be reduced by taking steps to prevent barriers to willingness becoming perceived barriers to ability.

## Background

The World Health Organisation describes an influenza pandemic as an event in which "a new influenza virus appears against which the human population has no immunity, resulting in several, simultaneous epidemics worldwide with enormous numbers of deaths and illness" [1]. In the United Kingdom (UK) the Department of Health (DH) is forecasting that up to half of the population could become infected with up to 750,000 deaths under the reasonable worst case scenario [2]. These assumptions work on the basis of cumulative clinical attack rates of up to 50%; 4% of symptomatic patients requiring hospital admission; and a case fatality rate of 0.2 – 2.5% [2]. Even at the lower end of these estimates, an influenza pandemic will place the National Health Service (NHS) under severe strain, and it is clear from the recent National Risk Register [3] that it is regarded as a significant threat to national security in the UK.

Healthcare workers (HCWs) will play a key role in any response to pandemic influenza, and will be in the frontline of exposure to infection. UK planning assumes that once a pandemic is confirmed, the NHS will "care for large numbers of cases, and will only provide essential care" for other patients [2]. Recent guidance, based on an (unreferenced) survey tool, suggests that up to 50% of the workforce may be absent from work at the peak of the pandemic because of caring responsibilities [4]. A modelling summary submitted to the DH by the Scientific Pandemic Influenza Advisory Committee Subgroup on Modelling estimates staff absenteeism at between 30–35% at the peak, taking into account the cumulative effect of staff illness, the need to look after ill children, and possible school closures [5].

It may not, however, be reasonable to assume that HCWs will be *willing *to work even if they are *able *to do so. For instance, during the early years of the Human Immunodeficiency Virus (HIV) epidemic doctors debated whether it was ethically permissible to refuse to treat those with HIV [6-10]; and during the 2003 Severe Acute Respiratory Syndrome (SARS) outbreak some HCWs were not willing to treat SARS patients [11-13]. HIV and SARS provide reasonable comparators to pandemic influenza, and it is not unreasonable, therefore, to assume that the response to pandemic influenza may be similar.

The limited data on factors influencing HCWs' willingness to work highlight a sense of professional obligation, estimated risk to oneself and ones' family and inclusion in preparedness planning [14-16] Ehrenstein and colleagues [17] found 28% of German HCWs (physicians, final year medical students, nurses and administrators) may abandon work in favour of protecting themselves and family. Qureshi and colleagues [18] found the most significant barrier to US HCWs' willingness to work was fear for their own and their family's health. A survey of clinical and non-clinical HCWs in the US estimated that up to 50% would be unwilling to work, with clinical staff more likely to attend than non-clinical [19]. Research from Singapore suggests that the risks posed to self and to family would be significant concerns for primary care physicians [20], and a similar Australian study of general practitioners highlights a strong sense of obligation to work coexisting with concerns about being provided with protective equipment and the welfare of dependants [16]. It cannot be taken for granted that these studies can be applied to workers from other health services nor that the results of these studies can be used to inform their attempts to modify attitudes ahead of a pandemic. Different countries have different health care systems and different healthcare cultures. Given that healthcare culture is likely to have an impact upon the willingness of HCWs to work, it is important that culture specific research is conducted.

UK emergency planning, and consequently patient care, will be improved if it is possible to establish the factors associated with UK HCWs' willingness to work, and identify the motivations HCWs have for continuing to work. This study, therefore, aimed to explore UK NHS HCWs' views about working during an influenza pandemic, in order to identify factors that might influence their willingness and ability to work and potential sources of any perceived duty to work. The majority of work in this area to date has utilised survey tools, and whilst large scale surveys can provide important and generalisable information about people's views and the frequency of those views, it is also important that qualitative research is conducted to begin to build a picture of why those views are held. Given the aim of research on the attitudes of HCWs towards working during pandemic influenza is to enable us to predict and modify behaviour, it is important to have data that will help us understand the 'why?' as well as the 'what?' and 'how many?'.

## Methods

### Setting

Participants were recruited from three NHS Trusts in the West Midlands, one acute teaching, one rural district general, and one Primary Care Trust.

### Ethical Approval

NRES approval for this project was granted by Nottingham Research Ethics Committee 2 (Ref: 07/H0408/120), and R&D approval was gained from each participating Trust.

### Recruitment

Recruitment took place using advertisements via internal e-mail, through managers and snowballing (using key participants to recruit others). All participants approached by managers were made aware that participation was voluntary, and managers would not be told who had eventually participated. All potential participants who contacted the research team, regardless of how they had heard about the study, were sent an information sheet, and those who were willing to participate were entered on a database. Participants for focus groups were purposively selected from this database according to job category with a view to getting as wide a range of HCW roles as possible, including a mix of age, gender and seniority. Signed informed consent was gained from all participants prior to participation.

### Design

Ten focus groups were planned with volunteer HCWs (grouped according to homogeneity of role: ×2 ancillary and clerical (A1 & A2), ×2 nurses (N1 & N2), professions allied to medicine (P), junior doctors (below consultant – JD), consultants (C), general practitioners (GP), managers (M) and community based HCWs (CH). Failure to identify a suitable time for a consultant doctor group led to interviews (n = 5) being undertaken with this category. Both focus groups and interviews followed a standardised topic guide, developed over a series of meetings of the authors and of the study steering committee. Open questions were used, enabling the content of the discussions, but not the form, to be participant led. The topic guide started by asking what participants knew about pandemic influenza, followed by questions about training and the effect they anticipated pandemic influenza to have on their work. Participants were asked if they felt they would be able to carry on 'business as usual as far as possible' [2] in their professional life; how likely they were to report to work if fit and well; whether they thought HCWs had a duty to work, why, and how far this duty extended. The discussions ended by asking participants to discuss the things that would worry them about working during an influenza pandemic, and what things they thought their employers, the Government, or professional bodies and unions could do to make it easier for them to work.

Focus groups are a recognised qualitative tool, suitable for research that aims to explore how people think and feel, where the emphasis is on exploring people's opinions, experiences, wishes and concerns [21,22] The dynamic, interactive, nature of focus groups means that participants are continually interacting with one another, exchanging views and perspectives, and this level of interaction enables the researcher to examine meanings, reasons and motivations "with a degree of complexity that is not typically available with other methods" (p16) [23] and to access the rich texture of normative influences on people's attitudes and behaviours [24]. The aim of a focus group is not to gather data that is statistically significant or generalisable to the wider population, but to gather information that can help us begin to understand why people may choose to act and behave in the way they do. For this reason, the results are not presented numerically, as in this context frequency has little meaning and detracts from the qualitative presentation style.

### Data analysis

All focus groups/interviews were transcribed *verbatim*, and were formally reviewed by three of the authors (SG/JI/JMP). Transcripts were initially free-coded by JI on ATLAS-ti software according to content [25], and then organised into thematic units that were continually re-visited and revised. Analysis and data collection occurred simultaneously, with a constant comparative method [26] utilised to ensure an iterative approach to the interpretive project. Analytic induction [25] was also employed, with themes being identified, hypotheses generated and fed into subsequent groups/interviews and then revised in the light of new data.

Given the nature of qualitative enquiry, the analysis was not conducted numerically. 'Significance' in qualitative research, and particularly in a focus group, cannot be determined by the frequency with which a view or opinion is raised, but rather in the manner in which it is raised, discussed, and negotiated by the group. What is important in a focus group analysis is not how many people stated a particular view, but rather what general themes emerged out of the discussion [27]. Bowling [28] points out that " [q]ualitative research describes in words rather than numbers" (p352), and as such, we have presented our results by describing themes that tended to arise out of the group process, rather than counting the number of individuals who expressed a particular view. This is justified, first, because the number of participants (n = 64) is insufficient to find any statistical significance. Second, our analysis focussed on what was said (the quality of views) rather than how often it was said (the quantity of views). What general frequency indicators are provided are given solely to give the reader an impression of whether the views under discussion were majority or minority views, and we have indicated in which groups the themes under discussion arose.

A different kind of study is needed to determine the prevalence of the views reported, and the qualitative data reported in this article have been used to develop a large scale survey tool, the work for which is ongoing [29]. This article only discusses our qualitative data, and focuses on the variety of views and perspectives present in our research population.

## Results

Of n = 64 participants, n = 32 were male, n = 31 female, and n = 1 undisclosed. N = 14 were aged 21–30, n = 12 were aged 31–50, n = 23 were aged 41–50, and n = 14 were aged 51 and over. N = 48 classified themselves as being White British, n = 3 as White Irish, n = 1 as White Scottish, n = 3 as Asian, n = 1 as Black African, n = 3 as Indian, n = 4 as other, and n = 1 undisclosed. N = 5 participants were junior hospital doctors; n = 5 were consultants; n = 12 were nurses; n = 6 worked in professions allied to medicine; n = 16 were ancillary workers; n = 10 were general practitioners, n = 5 were community healthcare workers; and n = 5 were managers. The demographic and professional spread of participants was not sought to be representative of NHS workers in the UK, but sought to access a wide range of views and perspective across a wide range of HCWs working in different areas of the NHS.

**Figure 1:**
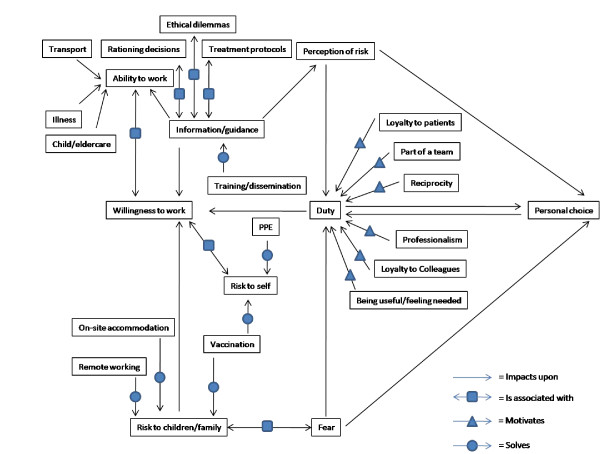
**Barriers to willingness and ability: associations and solutions**.

The overall themes that emerged are summarised in Figure 1. The network depicted expresses the relationships between the key themes that arose across all focus groups and interviews. Themes interact in one of four ways: (1) Impacting upon (a change in one may cause a change in the other); (2) Motivation (3) Association; (4) Solution.

In this paper we discuss key issues that relate to the 'duty to work' and 'barriers to working'. Eight main themes emerged relating to these two issues. Selected quotations are used to illustrate each of these eight themes.

### The Duty to Work

Overall, participants seemed to feel a strong sense of duty to work regardless of the circumstances and displayed a general willingness to work during an influenza pandemic. This sense of duty was found across all categories of workers, but was justified in a variety of ways that can be brought under three headings:

• a professional ethic

• a duty to help

• a work ethic and confederate loyalty

### A professional ethic (prominent in 4/9 focus groups and 5/5 interviews)

Some participants felt the duty to work is a professional duty that entails an obligation to work even in difficult and dangerous circumstances, because that is what they signed up for when they joined their profession. This kind of view was expressed most forcefully by hospital doctors and GPs, some of whom felt that this duty, which was developed during their training, was one owed both to their profession and to their patients. There were mixed views among participants about how far this obligation extended across different healthcare roles. Some doctors, for example, felt that the professional obligation did not apply so much to ancillary staff or nurses, though others felt that it applied to everyone, (see below).

#### Sense of duty developed during training

*JI: *So...what would you see as a source of this obligation to work, why would you personally feel that obligation to carry on? [long pause] It's a very tough question, I know.

*C4: *I suspect it must be something to do with the training, inherent to how it is being imparted. Now that's what I feel, I don't know what drives it to come to work, I suppose if I can come to work when I'm fit and well without a pandemic around, I expect that if I'm fit and well and I'm expected to work, then if it's a pandemic I'll have to come and work.

#### Duty confined to doctors

*JI*: There's various ways of looking at healthcare workers duties and some people will argue that health workers have a duty to tend to the sick and do their job no matter what. Some people will extend that to just doctors, some to doctors and nurses, and some would extend to anybody including admin and secretarial staff. Would you feel that that kind of duty does exist and how far do you think it extends?

*GP10*: Doctors yes, nurses probably, reception staff

*GP8*: No.

*GP10*: That depends on the people you've got. I think most of ours would turn up.

*JI*: What's the difference?

*GP10*: I just think it's the ethic. I mean you get into this job basically to look after people and, rather than man a phone for eight hours a day...

#### Duty to work extends to all roles

*JD2*: I think it's an obligation for doctors, I think that's set out by the GMC isn't it? It's the duty of a doctor.

*JD5*: It should be extended, I don't know, it's just my own opinion, it should be extended to everybody who comes in contact with the patient. Not only doctors, nurses as well

*JD2: *Well the hospital wouldn't function if it was just the doctors putting in the extra hours because you need support from the nursing staff, even down to the cleaning.

### A duty to help (prominent in 8/9 focus groups and 2/5 interviews)

Some participants across a range of jobs spoke about an obligation arising from a general 'duty to help'. Some, for example, claimed that being in a position to help another person was a sufficient motivator for helping them. Others expressed the view that if a person or a society is in need, that need gives everyone a moral incentive to take steps to meet that need. Notions of a duty to help were sometimes accompanied by references to the "Blitz/Dunkirk' spirit" of wartime Britain in the 1940s, and the belief that in the event of a social crisis we all have to pull together and do what we can for the common good. Here, the duty to help was constructed both as an individual and a social requirement to contribute to the common good, applying to us as persons and not *only *as HCWs. One key source of the duty to help for some HCWs was that they had specific skills that would make them particularly useful, and this seemed to make them feel they had a special responsibility where others might not, see below.

#### If you can help, you should help/The obligation to help those in need

*A1/9*: I am agency staff but I don't feel any different. If I'm of use then there's no point me sitting around redundant...when I can actually do something, It'd be more frustrating for me to be sat at home because I can't work even though I can contribute, it would just feel counter productive.

*********************************

*A1/2*: I kind of feel about it the same way as if I saw somebody get knocked over by a car, I'd go and help if I could.

*A1/6*: Yes, its human nature I think a lot of it.

A1/2: Yeah, that's the sort of way I feel about it. If there's something I could do, I would do it, you know. I think, I don't, I can't explain any more than that really, it's just that, you know

*A1/5*: And if you were in that situation you'd hope somebody would help you, you know

*A1/2*: Well exactly and I think it's the good of human kind really, that you wanna help if you can

#### Blitz/Dunkirk spirit

*N1/*5: We're British, Dunkirk spirit.

*N1/*3: Absolutely.

*N1/*5: And that has, bizarrely enough, that has been bandied around. You know that there are a lot of people that will just adopt this, well

*N1/*3: Spirit of the Blitz.

*N1/*5: Yeah, get on with it

#### Specific skills

*CH4*: I think it would depend on what help was required. If it was to administer medicines or injections, it's obvious which one would be needed more. If it was to sit there and just chat, support, wash then the nursing skills perhaps might be in use somewhere else so it's a really hard question to answer. I don't think you could just split it like that 'cause you'd need to know more wouldn't you? [everyone nods]

### A simple work ethic and confederate loyalty (prominent in 6/9 focus groups and 3/5 interviews)

Many participants, in a variety of roles, felt that absenteeism if one was able to work (whatever one's employment, in or out of the health service) was generally wrong and not just wrong during a pandemic.

This simple work ethic (see below) may be related to or reinforced by, a complex sense of loyalty and obligation to workplace confederates, in this case colleagues and patients. This account tended to emerge from a belief that, as both colleagues and patients depend upon you doing your job, by refusing to work (when you are physically able) you are letting both your patients and your colleagues down in way that is morally unacceptable, for example

#### Work ethic

*A2/8*: I mean personally, it don't matter what job I've got, whatever job I sign up for I look into that field and carry it on and I get, try to get personal satisfaction out of it and part of this becomes a dedication of where I want to be and what I want to do and it's like I'm now, I'm in domestics now and that gets some of my attention so therefore it's spread into that. So I'd come in anyway if I was all right, I would come in and that just ends it. Like if you had to do it anywhere else, you know you tend to go in because you think, 'Well someone else might be ill'.

********************************************

JI: If you were fit and well during an Influenza pandemic, how likely do you think it is that you would work as normal?

C3: Yeah sure, it's part of the role.

JI: Yeah, so I mean can you expand on that by 'part of the role'.

C3: Well that is part of what you do isn't it? You don't just work when the sun's shining.

*************************************************

HC3: You're not a nurse just when everything's okay, do you know what I mean? You're a nurse when things are not okay.

### Not wanting to let patients or colleagues down

*P4*: I feel like that really I would be letting those patients down if I didn't come in because they're my raison d'être, that's why I'm here.

*P1*: It's not just your patients. It's your colleagues as well.

*P5*: Yeah.

*P1*: You're letting your team down as well as the patients.

*P4*: That's right.

***************************************************

*N2/4*: I think that's the other thing. It's camaraderie. You get like a good little team on a ward and half the time if you do feel a little bit sort of a little bit sick you think 'oh well I've got to go in because they'll struggle without me'.

### Barriers to working

Although all participants tended to feel they have a duty to work during an influenza pandemic, there were nonetheless a number of perceived barriers to so doing. These tended to fall into one of two categories:

• perceived barriers to ability *(prominent in 9/9 focus groups and 5/5 interviews)*

• perceived barriers to willingness *(prominent in 9/9 focus groups and 5/5 interviews)*

These categories, however, were not clear cut in all cases, and the lines were most blurred when it came to concerns about childcare and family obligation. Some participants who had children regarded staying at home to look after them as a necessity that affected their ability to work, rather than a choice (see below). Childcare is not, however, obviously distinguishable from a barrier to willingness; for instance, where parents choose to look after their children themselves rather than rely on available others to do so for them. Choice, of course, is itself a nebulous concept. Where there is no externally available or accessible childcare, and where children are too young to care for themselves, there is a barrier to ability. However, what counts as 'accessible' or 'too young' and whether the available childcare is regarded as an adequate or acceptable alternative may owe as much to personal choice or preference as to inescapable circumstance.

It seems likely that the 'childcare' barrier is age and gender related. Women with young children tended to regard it as an insurmountable obstacle, with men raising the issue less often. Whether this gender difference was due to the participants' need to present themselves to their peers as participating in typical gender roles is unclear, although the discrepancy observed is predictable, and consistent with what we might expect. The group in which it was least prominent was the GP group, which was comprised largely of older men. The discussion was most prominent in the nursing groups, where women with young children were in the majority. The apparent lack of concern about this issue in the GP group may reflect gendered norms in the home, the age of the participants which meant that they were unlikely to have young children, or that GPs were better placed to afford reliable, private childcare. Similarly, the junior doctors did not discuss this issue: it was raised in passing only once. When they were questioned about why it had not been discussed, the answer was simply that they did not have children so it was not an issue.

For some, the duty to family was expressed as the simple claim that 'family comes first' which was taken for granted as an unassailable moral premise. If a child or a family member needed them they would not come into work pandemic or no pandemic: duties to families were more important than any duty to work. This was not so much a matter of weighing up competing obligations (to work and to family) but rather represented a pre-defined moral hierarchy acting as a barrier to ability (see below). For others, childcare functioned as a barrier to willingness, where a choice puts family before work.

Some barriers to ability were fairly concrete like being ill oneself and problems with transport (including lack of fuel). Participants recognised that they could not work if incapacitated by illness, and also anticipated that in the event of an influenza pandemic transport infrastructures might be affected, making it difficult to travel to work. Some participants thought that during an influenza pandemic people might be reluctant to use public transport for fear of becoming infected, leading to more people travelling to work in private cars. These same participants anticipated that Trusts would not have the parking space to accommodate additional demand, creating a further barrier for people who would otherwise be willing and able to work (see below). Insurmountable barriers to ability, however, exist at the extreme end of a continuum; for example where a person is literally too ill to get out of bed or function safely, where the person is infectious and poses a demonstrable risk to others, or where there is no fuel at all and the distance to work is too great to cover on foot or cycle. Further along this continuum, HCWs will be exercising choice about how to prioritise the different demands on their time and resources, and the greater the scope for preference or choice to be exercised, the more like a barrier to willingness the perceived obstacle becomes.

### Child-care as a barrier to ability

*N2/1*: Well I've got two small children, small children are probably more likely to get these things so, the ones at school, if the schools are closed there's no way I'd be able to come in because you can't [Laughs] You can't just leave your kids. They're obviously a priority.

****************************************************

*A1/5*: you'd have other outside influences wouldn't you? If you had children, if the schools were closed down and you'd got no-one to look after your children you... what, what options would you have then? You know your first duty is to their care so you would have to think about them before you could come in. If you'd got nobody to look after your children or equally your elderly parents if all that starts.

****************************************************

*P2*: I was gonna say where, in my case there's one adult to look after a number of children at home and I'm their sole means of support. And so to me they need that support every day you know whether the hospital needs mine or not, I'm not. You know that's the kind of a theoretical benefit to the hospital. You know 'cause I work in preventative health. So it's easy for me to say my kids need me every day, and it's gonna be a different decision for everybody isn't it?

#### Family comes first

*N2/1*: But it's not just people with children, it's people with old parents or you know it's home situations isn't it? It's family situations and family comes before anything.

#### Concrete barriers to ability

A1/2: I think there's a Government issue as well like, you know for example if I come on, I come to work on the train, if the trains were all down because there's no staff to run them or whatever and I have to come to work in my car, um where do I park, um, and then you, I mean the icing on the cake would be if you parked up, they just said park anywhere and then get a bloomin' ticket from Q-park

******************************************************

JI: What kind of effect do you think that Pandemic Influenza would have your work place and on your job?

C1: I think I would see it in two ways, one is if I'm personally myself infected, and affected then it would have an implication from the point of view of my ability to work, and if it's going to be infectious then clearly there's going to be the issue of isolation. So if I'm infected and if I'm isolated then I wouldn't be able to work. If I'm not infected and I need to come to work it would pose a question in my mind whether I'm going to get infected if I come to work.

### Negotiating risk and duty (prominent in 9/9 focus groups and 4/5 interviews)

Other individuals saw the duty to their family as one of many competing claims. Concern about taking the virus home and infecting one's family, for example, was not perceived as a barrier to ability when participants believed that they were in a position to negate or mitigate the risk by employing infection control measures, or minimising direct contact with family members by staying at the workplace or sleeping in the spare room. Some groups, however, particularly consultants and managers, felt that anyone who thought that absenteeism would reduce the risk to their family had failed to appreciate that 'pandemic' meant that the virus was endemic in the community (see below).

#### Negotiating risk and duty

GP5: So if it is that there is a substantial risk that you yourself may succumb which sort of people might not be too concerned about their own mortality but the young children and dependents might be more of a concern. Then you'd want to make sure that if you're gonna put yourself in the front line then it's something worth doing. So there's no point, there's no point and if you're gonna be there you ought to be very well co-ordinated nationally and so you know what you're doing. Get as much protection as you can.

JI: Would that, do you think, be a requirement of your going into work, or would you go in anyway?

GP5: I think most of us would go in, even me who's raising all these concerns, I think most of us would go in, but there's a little bit of me that says that I'd tell you on the day.

GP3: I think if it was gonna kill you, you know sort of people, you know sort of healthy people or you know in their sort of thirties or whatever like me supposedly, yeah it would be nice to think you were gonna have some sort of protection, some sort of pull them down type fancy mask and yeah I might sort of sleep in the shed rather than give it to my daughter, but yeah.

#### Understanding 'pandemic'

JI: So people have said 'Well it's not me I'm worried about, it's taking it home to my children'. I think given what you've said, that's not going to be a concern for you?

C2: Well I mean that seems ridiculous to me, I mean I just think the whole point about a pandemic is it affects the whole herd, the whole tribe and why should I worry about taking it home when they're more likely to catch it from school or shopping or, and I don't know whether that's right but that's the way I've always looked at it really...I think it's probably a false assumption that you're gonna keep them safe by not going to work.

### The risk to self (prominent in 9/9 focus groups and 2/5 interviews)

Despite disagreement on what the risk was and how it could be managed, the risk to family members of working was important to everyone whereas the risk to self seemed to be of less concern. Even when one GP broke the consensus that seemed to be emerging from her group by suggesting that if the risk was great she would not work, she made only passing reference to her own safety and quickly justified her position with reference to the safety of her family (see below). However, participants' apparent ambivalence to personal risk was to some extent belied by regular discussion of personal protective equipment (PPE). Whilst participants were reluctant to say outright that personal risk concerned them, many stressed the importance of being provided with effective PPE (for example see below).

#### Risk to self

GP2: Okay, can I be honest? If there was an outbreak I don't think I'd come in...If I put my life and my family's life at risk it's easier to say you would come in but then when you see your colleagues dying I think it's a different thing if you're taking it back to your family because you love them don't you, and I don't think if there was an outbreak and I saw my colleagues really sick on death's door that I'd want to be coming into work and putting my husband and my family at risk. I just don't think I could do it.

#### The need for PPE

N2/4: I think as well being aware of um showing, you know, how they use those paper masks in theatre whether that would be an effective barrier against it or whether you'd need something sort of

N2/3: The proper equipment, rather than a cheaper alternative which they tend to do don't they as well? Proper equipment would be best

N2/6: Yeah, what protection are the staff that's coming to work going to have against catching it from the patients?

### Reciprocity (prominent in 8/9 focus groups and 1/5 interviews)

The belief that the relationship between HCW and employer is not reciprocal was one of the most significant barriers to willingness. Specifically, participants did not believe that the efforts of HCWs would be reciprocated or rewarded. This was expressed in a variety of different ways, including an expectation that HCWs would get no thanks or recognition for their efforts, the worry that any PPE provided would be the 'cheaper alternative' (see above) and concern that workers would receive little guidance or decision-making support (e.g. with respect to how resources should be allocated, how treatment should be allocated, or whether decisions made would receive the backing of the Trust).

The majority of participants said they had been given neither information about pandemic influenza, nor been made aware of what would be expected of them during such a crisis, and this gave many the impression that their employing Trust did not care about them or take their needs seriously. Lack of information was a key theme across all groups, with the majority finding the lack of information and engagement a demotivator to work, while clear information, guidance and support seemed to be important motivators (see below). The obvious exception was the management group, which included public health doctors, who were concerned about giving staff too much information, as they did not know if current information was accurate. They reasoned that as it was so difficult to get information through to the workforce, it was a waste of resources to attempt to do so, and possibly counter-productive, if the information given turned out to be inaccurate. Their preference was to disseminate information if, when and as it was needed (for example below). This was in direct contrast to the views expressed by the majority of other groups, who wanted information immediately and to be involved in the planning effort. The GPs were most similar to the managers; most seemed confident that they could cope with the pandemic if and when it occurred, and believed that the necessary information and guidance would be sent to them as and when appropriate, and that their role was one of implementation.

#### The need for information

*N1/1: *It's giving people information as well, if you're more informed about something you're more likely to do it than if you get up and you're not told anything, why should I put myself at risk if I don't have all of the information?

#### The dangers of giving information

M6: I think one of the difficulties is that there was a lot of changes initially in the national guidance on what could be expected, and there was a reluctance to pass that information down and then have to review it, and you know that affects the creditability of the information they're receiving

M3: but it's still very difficult given that we don't know when or what to give information. And if you think about giving information down to that level, it so rarely gets down there that if you got it down there now and it was wrong you'd have very great difficulty changing it when you need it to be right. So it's not about keeping ignorant, it's about informing people when you know. And it would be, I think it would be very wrong to give detailed guidance when we don't know what we're or what, you know, what the dangers are. But we've got to have a system in place to give guidance in an authoritative manner when the time comes.

Both clinical and non-clinical participants were worried about being asked to perform a role they had not been trained for, and had concerns both about being a danger to patients and being subject to litigation if something went wrong. Participants tended to feel they would need more support than usual from their managers (to help them to make decisions, to fight their corner, and to give hands-on assistance). There was also a belief, however, that there would be less support during a pandemic. It was clear that many participants would be reluctant to take on extended roles without some assurance that they would be protected from litigation and without explicit guidance on how to negotiate the ethical dilemmas that the pandemic was likely to produce (see below).

#### Litigation worries

A2/8: One thing that crossed my mind, is if I'm called in to help, clinical-wise and I make a gaffe, and the relatives take me to court who's gonna protect me? And it'll make me decide whether I was gonna help or not really.

*********************************************

N1/5: There isn't enough, from what the Critical Care Community, are saying coming out of the Department of Health to reassure them and there's a big fear amongst medical staff that you know, two years down the line they'll be litigated against because they rationed and denied services to people because there weren't enough beds or because they chose one patient over another. And these are genuine concerns that are being expressed by nursing staff alone.

#### Apprehension about ethical dilemmas

CH1: Well who would get the vaccination, how would you choose?

CH5: I guess the consultant makes the decision who gets it and who doesn't.

CH3: So selection, like Auschwitz isn't it?

CH1: I wouldn't like to be...

CH?: That would be awful wouldn't it

CH5: I wouldn't like to think they'd think it would be left up to me......as an administrator...

CH4: Who you prioritise and deny people as well

CH5: I would be very reluctant to go anywhere without proper guidelines, because that would stay on your conscience forever wouldn't it.

#### Need for reciprocal support

P6: It is, it is a different decision for everybody but I think, personally I would be... I'd feel obliged to come in anyway professionally but I would like to know that the Trust doesn't – how shall I put this? – I would like to know that the Trust can rely on me but at the same time I can rely on the Trust to make sure of my safety...Because I'd feel obliged to come in and I will come in but they've got to make sure that they go the full mile as well.

A further, connected, issue was that of the general erosion of morale and goodwill in the NHS as a whole. This was connected to the previously mentioned expectation that the HCWs' role during a pandemic would not be appreciated, recognised or rewarded. Some participants believed that NHS staff generally felt so under-valued and under-appreciated that some would be unlikely to report for work if they thought they were at personal risk. The majority seemed to feel that, in a crisis, their sense of obligation to their patients or colleagues would overcome their generally low morale, but anticipated that many of their colleagues would not feel the same way (see below).

#### Wanting to feel appreciated

A1/8: If they just show they're grateful for what you did. Say if a patient says to me, 'Oh you've kept my room spotless while I've been in here', it gives me a boost to think I've done something, but when you don't get no credit, then that's a knock back to you. That's when morale goes down.

A1/5: Is it a matter of esteem as well isn't it, you know?

A1/1: Come the end of the pandemic you've kept your bone marrow transplant ward isolated, clean and none of the patients have come down with the flu or whatever it is, and everything goes in the paper and you don't get a single mention that's gonna be soul destroying

#### Erosion of goodwill/morale

N2/4: I think for years now a lot of the NHS has run on goodwill of nurses and I think that bit by bit, especially the Trust where I come from...that's why I left there, the goodwill was eroded...

N2/1: They beat it out of you don't they? ...You're right. They do! They badger you and badger you until...I mean it's a caring profession. It's not a job you do for the money. It's a job you do because you want to do it but there is a limit...

#### Some people will not work

CH5: I mean I could probably split my staff in two camps, of the administration camp there's those I know would be there and they'd give their time and those that would say 'Sorry, no'. And that's just because of the people that they are and then the other obligations that they have.

## Discussion

The NHS staff in our study, across all roles and professions, tended to believe that they should, and would, work through an influenza pandemic. There was a widespread belief that they had a duty to work, and that in not continuing to work they would be doing something morally wrong. The basis for this sense of duty varied from group to group. Some were motivated by a sense of professional obligation; some by a general duty to help those in need; and others by a work ethic or feelings of confederate loyalty

Despite this, there were also barriers to working that impacted on this sense of duty, prominent amongst which were the need to care for/protect dependents and lack of information, guidance and support. These can be either barriers to ability or barriers to willingness, although the two are not always readily distinguishable. Most barriers seem to form a continuum with preference at one end and insurmountable circumstance at the other with increasingly difficult choices in the middle. The harder the choice, the more likely it is to be perceived as a barrier to ability; for instance, choosing to walk into work will be easier if it takes half an hour and if one is relatively used to exercise than if it takes an hour and a half and one is not. In the latter case, the absence of motorised transportation is more likely to be perceived as a barrier to ability. Equally, it would be wrong to assume, however, that it is always possible in practice to make a choice that is present in principle; a position that fails to appreciate the impact of inflexible external constraints and social circumstances. A decision to work (or not to work) is likely to be the result of a combination of motivations and beliefs, which interact with both genuine and constructed barriers to ability. For some, this combination may result in a genuine barrier to ability or a barrier to willingness that is genuinely perceived as a barrier to ability, and for others simply a barrier to willingness.

Which of these motivations, and which of these barriers, proves to be the most significant in the event of an influenza pandemic is something that can only be known and understood after the event. The barriers to, and motivations for, working, however, that we have identified may suggest forms of remedial action if these barriers and motivations are found to be prevalent in the workforce. The key to the efficacy of this remedial action may be to effect changes that prevent barriers to willingness from becoming insurmountable barriers to ability. If HCWs are concerned about infecting their families then steps might be taken to minimise this risk. Similarly, if transport is a likely issue steps might be taken to facilitate transport to and from work. Ensuring that staff are protected from litigation, and ensuring that they *know *they are protected, may also remove a barrier to taking on extended roles. This seems to be in line with DH guidance that staff should be provided with appropriate indemnity [30], although the definition of negligence found in the DH Human Resources guidance implies that no special protections will be given to staff working in extended roles in an emergency situation [4]. If we are correct in hypothesising that many barriers to working lie along an ability/willingness continuum, the key to effective mitigation is likely to be taking steps to tip the scale so that more barriers than not are experienced as barriers to willingness – which are more negotiable than barriers to ability. This will at least ensure that more HCWs than not feel they are in a position where working is an option for them.

One concern prominant in all groups (with the exception of the doctors and managers – who seemed to feel they knew what they supposed to do) was that participants felt they were not being told what was expected of them. If this is a widespread problem, one effective strategy (and one already indicated in DH planning guidance [30]) may be to have a policy of education and communicating emergency plans to staff, outlining what is known, what is not known, and what is expected of them. If Trusts are concerned about disseminating information before all the facts are known, then a policy of 'explicit uncertainty' might be adopted. The key point is that HCWs may not necessarily expect to be told all the answers, but they want to be kept in the loop and to be reassured that when information becomes available it will be communicated to them. Simply ensuring that systems are in place for the dissemination of information when it becomes available is not enough. The existence of these systems may have to be more effectively communicated to encourage the feeling that the needs of workers are being acknowledged. Our data suggests that giving workers evidence in the pre-pandemic phase that goodwill expended by them during a pandemic will be reciprocated by employers will encourage that expenditure. Reciprocity was identified as a key factor in the ethical guidance published by the DH [31], and this study reinforces its significance – suggesting that reciprocity is not just an ethical concern, but also a very practical one. Building goodwill amongst staff and encouraging confederate loyalty is likely to be an effective strategy to increase the motivation to work amongst HCWs. This is, however, no small task and it is unlikely that simply telling staff they are needed and are appreciated will be effective. A more promising avenue may be to encourage team cohesion in small units. It has been observed that soldiers on active duty may be motivated more by feelings of in-group loyalty than by a commitment to abstract ideals [32] (although a causal link between team cohesion and effectiveness/performance has been challenged [33]). It seems likely that similar team motivations may be at work with HCWs. Feelings of team cohesion may not enhance an HCW's effectiveness, but it may deter absenteeism.

A significant strength of this study is that participants were sampled from three different NHS settings across a wide variety of roles, and yet the overarching themes were consistent across groups. The findings also concord with previous work conducted overseas, showing some resonance with the findings of both Qureshi *et al *[18] and Ehrenstein *et al *[17] insofar as the perceived risks to family and children were very prominent. As one reviewer of this article pointed out, this suggests that barriers to working may be similar across some nations, and indicates there may be some benefit to the international sharing of strategies for dealing with HCW absenteeism.

An obvious limitation to these findings is responder bias. Participants in a focus group or interview may be the type of people who are already motivated and interested, or feel strongly about the topic area. Since it is likely that such individuals would be more willing to work during an influenza pandemic, our results may overstate HCWs' willingness to work. Another limitation to this study is the responder bias towards people identifying themselves as 'white British'. Further work, focussing on minority groups, may be required if there is reason to think that the views of white British people are likely to be different to those of other ethnic groups. However, a specific analysis looking at the views of our non-'white British' participants showed that no such difference was visible within our focus groups.

## Conclusion

This study attempted neither to provide estimates of the proportion of NHS staff who may work in the event on an influenza pandemic nor to predict the characteristics of such staff, though this work is ongoing [29]. It does, however, suggest that although UK HCWs may feel a general obligation to work during an influenza pandemic, there are a number of possible barriers to working that may significantly reduce the workforce of the NHS at such a time. Some of these barriers may be insurmountable but others may not. Relatively simple steps could be taken that might increase the likelihood of HCWs being willing and able to continue to work.

Current UK planning [4] assumes that up to 50% of the NHS workforce may require time off at the peak of a pandemic. Given that this level of absenteeism is likely to cause significant problems for a health service already in crisis, it is important that every means of encouraging HCWs to work if they are able are identified. The data presented in this article suggests a number of factors that may affect HCW's willingness to work. Further, if, as we suggest, factors affecting ability and willingness lie along a continuum, it will be important to take measures to prevent barriers to willingness becoming perceived barriers to ability. Where barriers to working are based on deeply held moral values – such as that of putting family or more specifically children first – such barriers may prove as insurmountable as being too ill to work. More effective might be efforts to ensure that wherever HCWs feel that a choice to work or not is necessary or possible they are inclined to choose in favour of working. They are more likely to be so inclined if they continue to perceive whatever barrier gave rise to the choice as one of willingness rather than ability. Based on our focus groups with UK HCWs, we have suggested a number of possible measures that may achieve this which include providing transport, accommodation and useful and timely information to staff as well as demonstrating to them that they are needed and valued. These, or similar, measures may encourage the view that choosing to work is a realistic and acceptable option. If people feel they have no choice but to stay away, they will stay away. If people find that there are no concrete barriers that effectively prevent them from being able to work, they have to make the choice for themselves, and where this choice is available other factors such as perceptions of duty (however conceived), peer pressure, or the knowledge that they will be supported and thanked may provide the motivation to make the choice in favour of working.

## Abbreviations

HCW(s): Health Care Worker(s); DH: Department of Health; WHO: World Health Organisation; NRES: National Research Ethics Service; NHS: National Health Service.

## Competing interests

The authors declare that they have no competing interests.

## Authors' contributions

All authors were involved in the design of the project. HD was Principle Investigator, with the SG and JMP acting as qualitative supervisors (and SW as quantitative supervisor). Focus groups and interviews were organised and conducted by JI, with assistance from HD, TS and JMP. Primary analysis was conducted by JI, and formally reviewed by SG, JMP and JIP, with additional input from HD and CG. The first draft of this manuscript was produced by JI, which was then reviewed and revised by all authors. All authors read and approved the final manuscript.

## Pre-publication history

The pre-publication history for this paper can be accessed here:


